# Intraarticular bone grafting in atlantoaxial facet joints via a posterior approach: nonstructural or structural—a minimum 24-month follow-up

**DOI:** 10.1186/s13018-021-02630-z

**Published:** 2021-08-23

**Authors:** Jun Zhu, Jian Wu, Keyu Luo, Zhong Wang, Huaijian Jin, Yufei Jin, Yingbo Wang, Mingyong Liu, Peng Liu

**Affiliations:** 1 Division of Spine Surgery, Department of Orthopedics, Daping Hospital of Army Medical University, Chongqing, 400042 China; 2 State Key Laboratory of Trauma: Burns & Combined Wound, Institute for Traffic Medicine of Army Medical University, No. 10, Changjiangzhilu, Daping Street, Yuzhong District, Chongqing, 400042 China

**Keywords:** Bone grafting, Fusion, Atlantoaxial complex, Facet joint, Reduction

## Abstract

**Objective:**

To investigate the necessity of nonstructural or structural intraarticular bone grafting in atlantoaxial facet joints via a posterior approach and the influence by the presence of basilar invagination (BI).

**Methods:**

From November 2016 to October 2018, patients who underwent posterior atlantoaxial or occipitocervical arthrodesis surgery at one institute were retrospectively reviewed. Operation records, preoperative and postoperative clinical status, and radiological films were analyzed.

**Results:**

Thirty-three patients (19 without BI, 14 with BI) underwent posterior facet joint release followed by intraarticular bone grafting were enrolled finally. Twenty-four nonstructural (15 without BI, 9 with BI) and 9 structural (4 without BI, 5 with BI) grafting were performed. The average follow-up was 32.15±6.73 months (24–47 months). Among them, 1 (3.03%) implant failure occurred, and 32 (96.97%) achieved satisfactory neurological outcomes, including 28 (84.85%) complete and 4 (12.12%) acceptable reductions with complete fusion within 6 months. For patients without BI, structural and nonstructural grafting showed no significant difference in terms of reduction maintenance (100% vs 73.33%, *p* = 0.530), while for those with BI, structural grafting significantly increased the postoperative height of the joint space (5.67±1.22 mm vs 3.43±1.78 mm, *p* = 0.002) and maintained it much better than nonstructural grafting (88.89% vs 20.00%, *p* = 0.023), contributing notably to BI correction.

**Conclusion:**

Intraarticular structural bone grafting in atlantoaxial facet joints has the advantage of maintaining anterior column height in the case of lateral mass collapse or when BI correction is needed; otherwise, nonstructural bone grafting is enough.

**Supplementary Information:**

The online version contains supplementary material available at 10.1186/s13018-021-02630-z.

## Introduction

Atlantoaxial arthrodesis is indicated in cases of instability, dislocation, infection, and other etiologies involving the upper cervical spine. Posterior [1, 2], anterior [3], and combined approaches [4] could be employed, among which the posterior approach is the most frequently employed approach, followed by the anterior and combined approaches. Compared with the era of Gallie [5] and Brooks [6], the fusion rate has improved significantly since Magerl [7, 8], Goel [1], Harms [2], and other rigid internal fixation techniques were introduced for atlantoaxial arthrodesis [9].

Successful spinal fusion requires a well-prepared grafting bed, an ideal grafting material owing property of osteogenesis, osteoinduction, and an appropriate local biomechanical environment [10, 11]. Traditionally, the grafting bed is most frequently located at the posterior arch of C1-2 [4, 7, 8, 12]. In the past two decades, an increasing number of studies have reported bone grafting in the facet joint space of the atlantoaxial complex as an alternative to or in combination with traditional techniques [13–16]. Most case series reported that a cage [13–17], a custom-made titanium spacer or the corticocancellous bone, was inserted into the facet joint space as grafting material, which gave the impression that structural bone grafting was required for such situations. However, it remains in question whether it is mandatory. To address this issue, we retrospectively analyzed a case series containing both nonstructural and structural bone grafting. The aims of the present study were to investigate the intraoperative feasibility of structural bone grafting, to investigate the indications of both grafting measures and to evaluate the clinical and radiological outcomes.

## Material and methods

### Study patients

This study is a retrospective review of 33 patients who underwent upper cervical spine surgery at a single institution from November 2016 to October 2018. Inclusion criteria: (1) posterior atlantoaxial arthrodesis indicated in the case of atlantoaxial instability or dislocation without congenital anomaly; (2) posterior occipitocervical arthrodesis indicated in the case of atlantoaxial instability, dislocation, or basilar invagination (BI) with a congenital bone anomaly; (3) posterior occipitocervical or atlantoaxial arthrodesis indicated in the case of lateral mass collapse and secondary BI due to rheumatoid arthritis; and (4) minimum follow-up of 24 months. Exclusion criteria: (1) the anterior approach was employed alone or in combination with the posterior approach; (2) the posterior approach was employed, and traditional posterior fusion was performed; however, the facet joints were not exposed for any reason; (3) posterior internal fixation was employed for temporary fixation of the fracture and without fusion; and (4) the duration of follow-up was less than 24 months.

This study was registered in the Clinical Trial Registry (ChiCTR2000038815) and in line with the Declaration of Helsinki. The study was approved by the Ethics Committee of our institute. Informed consent was obtained from the patients.

### Surgical procedure

All patients underwent surgery under general anesthesia in the prone position (reverse Trendelenburg). Gardner-Wells traction tongs were instituted following anesthesia. The weight of traction ranged from 3 to 10 kg according to the demands of the reduction and/or the body weight of the patients (maximal traction weight equals one fifth of the patient’s body weight). A midline posterior approach was employed, and an operating microscope was used. C1 lateral mass screws and C2 pars/pedicle screws were implanted according to Goel’s [1] and Harms’s [2] techniques. Polyaxial screws (Cobra II posterior cervical fixation system, KangHui Med., ChangZhou, People’s Republic of China) were used in all patients (length ranged from 20 to 32-mm diameter of 3.6 mm).

**Fig. 1:**
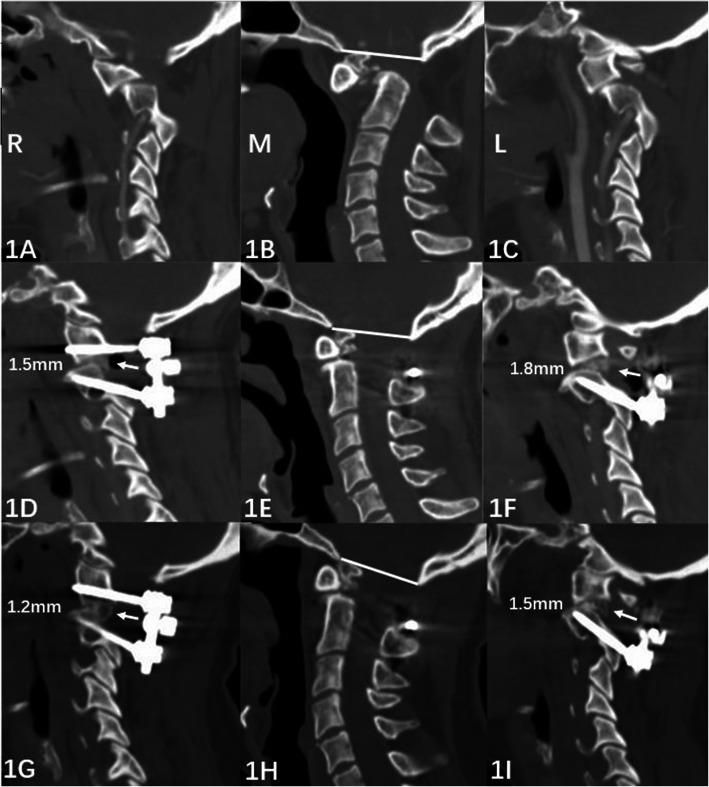
An atlantoaxial dislocation without BI managed with posterior nonstructural fusion. Female, 40 years. She sustained myelopathy (JOA score 11) due to atlantoaxial dislocation for 6 months and underwent atlantoaxial facet release, C1-2 fixation, reduction, and intraarticular nonstructural fusion via a posterior approach. At the final follow-up, her JOA score was 16. **A–C** Preoperative CT slice: right atlantoaxial facet joint (1A), midsagittal (1B, white line indicates Mcrea’s line), and left atlantoaxial facet joint (1C) views showed the relationship of C1 to C2, and there was no BI. **D**–**F** Immediate postoperative sagittal CT slice: nonstructural grafts (white arrow) within the atlantoaxial facet joints. The height of the joint space was 1.5 mm on the right side (1D) and 1.8 mm on the left side (1F). Midsagittal CT (1E) showed anatomical reduction of the dislocation. **G**–**I** CT slice at 6 months following surgery: grafts bridged the neighboring facet joints (white arrow) and suggested successful fusion. The height of the joint space was maintained well (1.2 mm on the right side, 1G; 1.5 mm on the left side, 1I). The reduction was maintained well (IH)

**Fig. 2:**
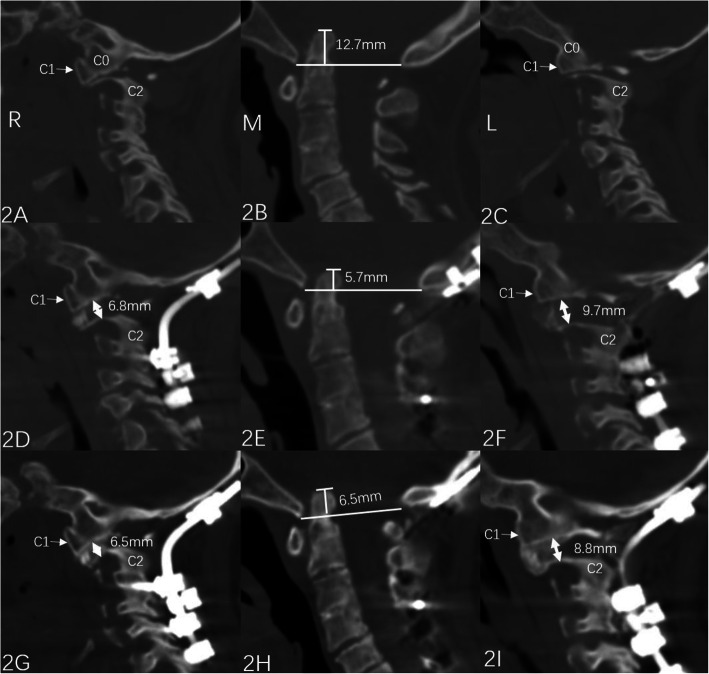
A rheumatoid arthritis with BI managed with posterior structural fusion. Female, 41 years. She reported a history of rheumatoid arthritis for 16 years. Symptoms of myelopathy lasted for 1 year (JOA score 9). She underwent occipital-C4 fixation, release of the atlantoaxial facets, and intraarticular structural fusion. Her JOA score improved to 15 at the final follow-up. **A**–**C** Preoperative CT slice: The right atlantoaxial facet joint (2A), midsagittal (2B), and left atlantoaxial facet joint (2C) views showed the relationship among C0, C1, and C2. The distance from the tip of the odontoid process to Mcrea’s line was 12.7 mm, and the secondary BI was due to RA-induced collapse of the lateral mass column. **D**–**F** Immediate postoperative CT slice: Structural grafts were inserted into facet joints with heights of 6.8 mm on the right side (2D) and 9.7 mm on the left side (2F, white double arrow), and the BI was lessened to 5.7 mm (2E). **G**–**I** CT at 5 months following surgery: Both the BI correction (6.5 mm, 2H) and the height of the joint space (6.5 mm at right, 2G; 8.5 mm at left, 2I) were maintained well, and robust interfacet fusion was acquired

The autograft was harvested at the posterior iliac crest. Nonstructural grafts were prepared according to Wangchao’s [4] (Fig. 1A–I). A structural graft was prepared as a tricortical graft, and the size of the graft was modified to fit the gap between the facet joints (Fig. 2A–I). Whether nonstructural or structural grafting was selected was analyzed according to the operation records, video recordings, and discussion with the surgeons. Autogenous nonstructural grafting between the C1 posterior arch and C2 lamina following meticulous decortication [4, 12] was employed as a standard procedure in all patients.

### Clinical and radiological evaluation

Radiological evaluation included preoperative, postoperative, and follow-up X-rays, CT, and MRI. The main indexes included the clivus-axial angle and the height of the lateral mass joint space. Implant failure, reduction, and bone fusion were evaluated in postoperative radiological films. According to postoperative CT and MRI, the reduction of the dislocation was categorized as one of three conditions: (1) complete reduction in the sagittal and vertical planes; (2) acceptable incomplete reduction in any plane, as indirect neurological decompression is achieved by reduction; and (3) unacceptable incomplete reduction or reduction failure. Successful maintenance of reduction was defined as follows: at the last follow-up, the height of the lateral mass joint space had lost less than 1 mm or 50% of the primary acquired height on postoperative CT, and the clivus-axial angle had lost less than 5°. The bone fusion criteria were defined on CT scans as follows: (1) atlantoaxial facet fusion: within the facet joint space, trabeculae bridge the neighboring articular surface of the facets; and (2) posterior atlantoaxial fusion: nonstructural autografting bridges the posterior parts of the aimed segments [4, 12, 18]. Any clinical complications were recorded. Follow-up was performed at postoperative months 1, 3, 4, 6, and 12 in the outpatient clinic, and X-rays and/or CT and MRI examinations were performed.

### Statistical analysis

Continuous variables are presented as the means with standard deviations, and categorical variables are reported as frequencies (percentages). To compare proportions of two nominal variables, Fisher’s exact test of independence were used. Repetitive measurement deviation analysis was used to compare the variables at different observation times (preoperation, postoperation, and last follow-up). Means of continuous variables at the same observation point were compared by independent samples Student’s *T* test. Statistical analyses were performed using SPSS version 21.0 software (IBM Corp., Armonk, NY, USA). Significant differences were defined as those with *p* < 0.05.

## Results

### Patients’ cohort and characteristics

Forty-five patients who underwent upper cervical spine surgery were reviewed. Twelve were excluded according to the exclusion criteria, and 33 patients, including 14 males and 19 females, were enrolled in the study. There were three categories of diagnosis: (1) C1-2 instability or dislocation without a bony anomaly and without BI (*n*=19); (2) C1-2 instability or dislocation with assimilation of the atlas and/or congenital C2-3 unsegmentation with BI (*n*=11); (3) lateral mass column collapsed due to rheumatoid arthritis, which led to secondary BI (*n*=3). Therefore, there were 19 patients without BI and 14 with BI. Twenty-four patients underwent nonstructural grafting in facet joints, and 9 underwent structural grafting. There was no significant difference in general data between the two groups (Table 1). Two patients reported a history of previous surgery in the upper cervical spine, including one failed attempted reduction and fusion and one foramen magnum decompression without reduction.

**Table 1:** General information and clinical characteristics of patients with two grafting methods (*n*=33)

	Total	Nonstructural grafting (*n*=24)	Structural grafting (*n*=9)	*p* value
Age				0.246
Mean±SD	51.58±16.24	50.94±16.07	50.52±16.53	
Median (mix-max)	14-74	21-74	14-72	
Gender (*n*, %)				0.886
Male	14 (42.42)	10 (41.67)	4 (44.44)	
Female	19 (57.58)	14 (58.33)	5 (55.56)	
Basilar invagination (*n*, %)				0.442
Yes	14 (42.42)	9 (37.5)	5 (55.56)	
No	19 (57.58)	15 (62.5)	4 (44.44)	
Diagnosis (*n*, %)				0.348
1	19 (57.58)	15 (62.50)	4 (44.44)	
2	11 (33.33)	8 (33.33)	3 (33.33)	
3	3 (9.09)	1 (4.17)	2 (22.23)	
Operation time (min)	213.76±62.74	215.75±62.67	206.69±62.88	0.065
Intraoperative bleeding (ml)	148.64±93.92	150.78±94.60	152.76±98.05	0.409
Improvement in JOA score (%)				
1 day	11.96±13.92	11.17±13.35	13.23±14.29	0.594
1 month	43.33±28.50	43.51±28.93	46.09±27.92	0.989
3 months	59.01±29.70	59.29±30.13	60.54±29.45	0.683
6 months	74.27±26.80	74.25±27.23	72.35±27.73	0.893
Last follow-up	79.58±27.53	79.33±27.94	77.96±28.83	0.889
Complications (*n*, %)	13 (39.39)	9 (27.27)	4 (12.12)	0.509
Implant failure	1 (7.69)	1 (11.11)	0 (0)	
Occipital neuralgia	12 (92.31)	8 (98.89)	4 (100)	
Follow-up time	26.03±4.45	26.33±4.73	25.22±3.70	0.531

Diagnosis 1 means “C1-2 instability or dislocation without bony anomaly and without BI; Diagnosis 2 means “C1-2 instability or dislocation with assimilation of the atlas and/or congenital C2-3 unsegmentation with BI”; Diagnosis 3 means “lateral mass column collapsed due to rheumatoid arthritis, which lead to the secondary BI”

### Nonstructural or structural grafting selection

By reviewing the operation records and video, the surgeons in charge claimed that the technique choice depended on the height of the gap in the facet joints following release, debridement of lesions, excision of cartilage, and reduction of the dislocation. If the height of the gap ranged from 3 to 5 mm, nonstructural grafting was selected, and bone chips were inserted into the gap. If the height of the gap was more than 5 mm, structural grafting was selected.

### Clinical outcomes

Sixteen patients accepted atlantoaxial fusion was performed, and 17 patients accepted occipitoaxial fusion. One patient underwent foramen magnum decompression, while two patients underwent one-stage subaxial cervical laminoplasty due to stenosis. The average intraoperative bleeding was 148.64±93.92 ml (50–410 ml). No vertebral artery or spinal cord injury occurred. Preoperative, postoperative, and final neurological function were evaluated by the JOA score. Except for one patient, all those with neurological impairment showed significant improvement following surgery. The average improvement in JOA score was 11.96±13.92% at the first postoperative day, 43.33±28.50 % at 1 month, 59.01±29.70% at 3 months, 74.27±26.80% at 6 months, and 79.58±27.53% at the last follow-up. Major complications included one case of implant failure and complete reduction loss at the second month following surgery, who achieved solid posterior fusion at the fourth postoperative month, and was asked to undergo transoral decompression in the future. Minor complications included occipital neuralgia in 12 patients, 11 of which experienced spontaneous resolution within 3 months. One patient complained of persistent occipital neuralgia, which was relieved after implant removal surgery 12 months after the index operation (Table 1).

### Radiological outcomes

#### Reduction of the dislocation

Immediate postoperative radiological examinations showed complete reduction in 28 cases (84.85%), acceptable incomplete reduction in 4 cases (12.12%), and unacceptable reduction in 1 case (3.03%). At 2 months, the unacceptable reduction converted to reduction failure due to screw purchase loss at the occipital end.

#### Maintenance of the sagittal and vertical reduction

**Fig. 3:**
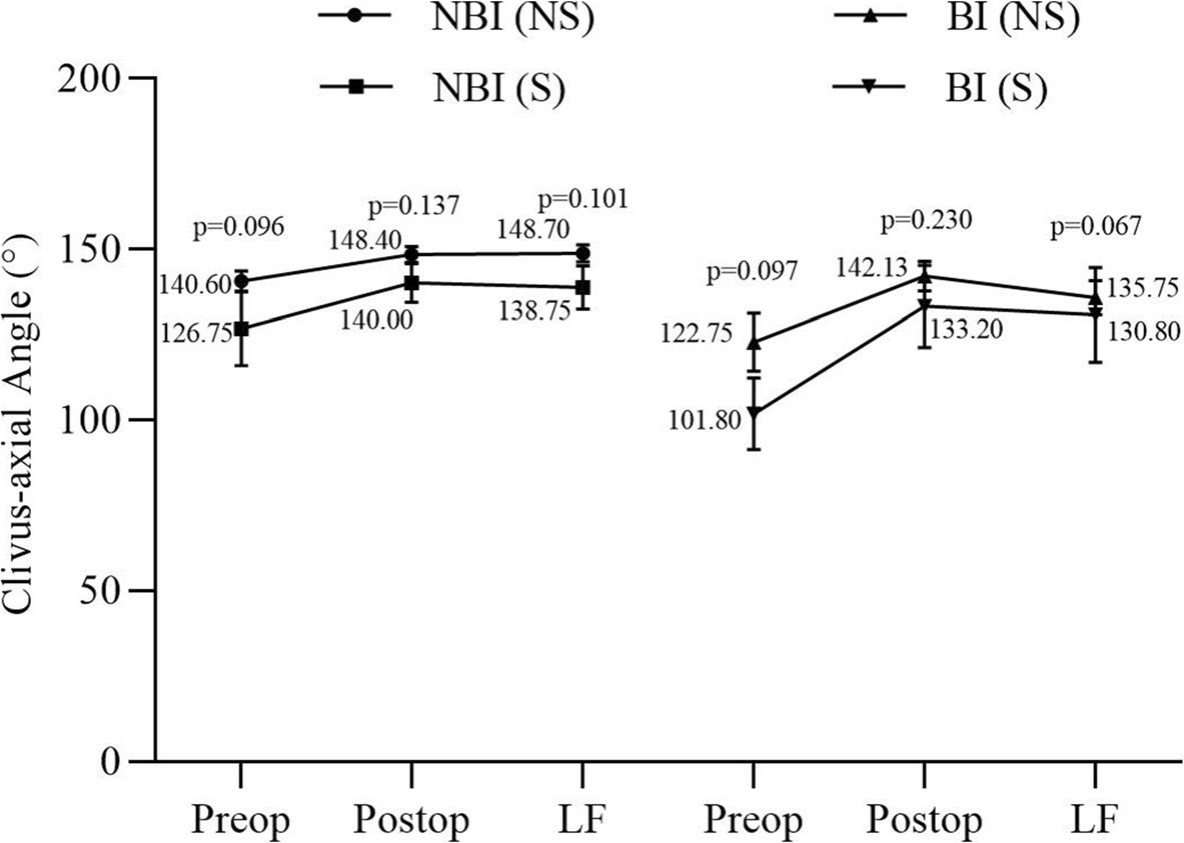
Impact of grafting measures on clivus-axial angle. Regarding the clivus-axial angle, two grafting measures showed no impact on clivus-axial angle correction, regardless of BI status. There was no significant difference in CA angle between the two groups at the same observation time point. BI, basilar invagination. NBI, no basilar invagination. S, structural grafting. NS, non-structural grafting. Preop, preoperation. Postop, postoperation. LF, last follow-up

**Fig. 4:**
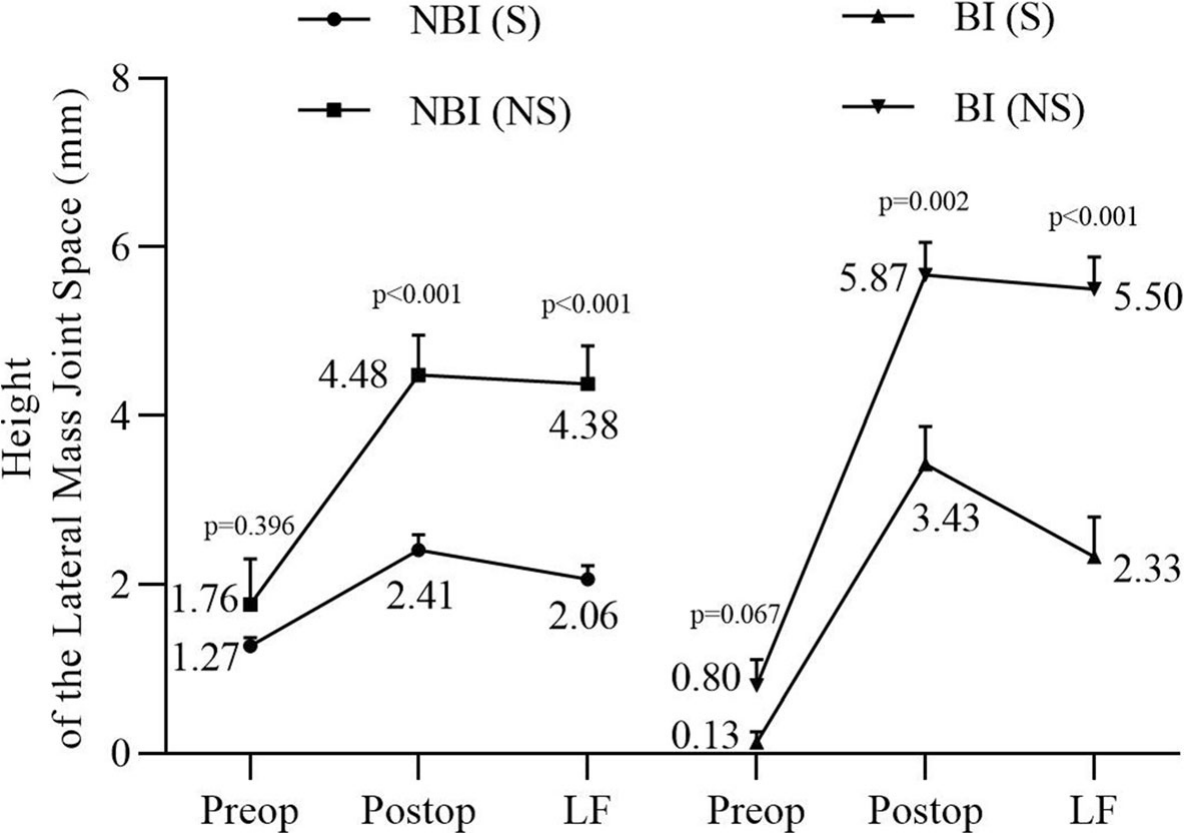
Impact of grafting measures on height of the lateral mass joint space. As the height of the lateral mass joint space, no significant difference in the preoperative index was detected between different grafting measure groups in patients with and without BI (*p* > 0.05), while the postoperative height was significantly different (*p* < 0.05). BI, basilar invagination. NBI, no basilar invagination. S, structural grafting. NS, non-structural grafting. Preop, preoperation. Postop, postoperation. LF, last follow-up

The clivus-axial angle and height of the atlantoaxial facet joint space were measured preoperatively, postoperatively, and at the last follow-up. Thirty-two patients with satisfactory clinical outcomes were enrolled finally. For patients without BI, grafting measures provided a similar rate of successful maintenance of the reduction (*p* > 0.05), while for those with BI, structural grafting provided significantly better reduction maintenance rates than nonstructural grafting (*p* < 0.05, Table 2). Regarding the clivus-axial angle, two grafting measures showed no impact on clivus-axial angle correction, regardless of BI status (Fig. 3). As the height of the lateral mass joint space, no significant difference in the preoperative index was detected between different grafting measure groups in patients with and without BI (*p* > 0.05), while the postoperative height was significantly different (*p* < 0.05), which indicated that structural grafting could significantly increase the height of the lateral mass joint space regardless of the existence of BI. The height at last follow-up and height loss showed similar differences (p < 0.05), suggesting the advantage of structural grafting owing to the maintenance of the expected vertical reduction (Fig. 4).

**Table 2:** Impact of grafting measures on successful maintenance of the reduction

BI	Grafting	*n*	Successful maintenance	Unsuccessful maintenance	*p* value
No	NS	15	11 (73.33%)	4 (26.67%)	0.530
	S	4	4 (100%)	0 (0)	
Yes	NS	9	1 (11.11%)	8 (88.89%)	0.023
	S	5	4 (80%)	1 (20%)	

*BI* basilar invagination, *NS* nonstructural grafting, *S* structural grafting

#### Evaluation of bone fusion

Solid posterior fusion was achieved within 3–6 months postoperatively in 31 patients, while one patient did not have a satisfactory fusion mass until 12 months, but no implant failure has been detected to date. Intraarticular bone absorption was observed in one patient in the nonstructural bone grafting group, which was regarded as the result of insufficient removal of cartilage. Twenty-three cases of nonstructural bone grafting and 9 cases of structural bone grafting achieved the anticipated C1-2 lateral mass fusion.

## Discussion

Goel and Laheri [1] first reported techniques for exposure of the posterior aspect of the C1-2 facet joints and C1 lateral mass screw insertion. Harms and Melcher [2] made the technique popular worldwide. In 2004, Goel [14] inserted a titanium spacer into the facet joint space with the aim of sagittal and vertical reduction in cases with basilar invagination. Thereafter, similar techniques were reported more frequently, and in most reports, a hydroxyapatite block, a titanium or PEEK cage packed with autogenous or the allogenous bone, was employed as the strut graft material [15–17, 19–21]. However, we considered whether intraarticular bone grafting was indicated and whether this should be considered before applying the technique in every surgery uniformly. Not every posterior surgery in the craniocervical junction should expose and release the atlantoaxial facet joints, such as in temporary fixation for upper cervical fractures and stabilization surgery for reducible atlantoaxial dislocation. For reducible instability or dislocation, treatment should be confined to internal fixation and posterior fusion, which is sufficient for achieving the goals of reduction and stability. The present study excluded these patients because we considered intraarticular bone grafting in facet joints to not be indicated. The enrolled patients exclusively underwent the release of the facet joints, which enabled the irreducible dislocation to be completely or partially reduced [4, 12–14, 22, 23]. Intraarticular grafting within facet joints followed the procedures of internal fixation and reduction, which provided an additional grafting bed and promoted the possibility of fusion [13–16]. In rare cases, intraarticular grafting is the sole choice because the posterior fusion bed is not available due to foramen magnum decompression.

According to our surgical experience, release of atlantoaxial facet joints is indicated when acceptable reduction of a dislocation is not acquired by sufficient skull traction. Dissection of tissue hindering a reduction and forceful levering of the C1 lateral mass by an elevator could mobilize the atlantoaxial joints, thereby converting an irreducible dislocation into a completely or partially reducible one. As an augment for the posterior fusion, the admirable clinical and radiological outcome suggested that intraarticular joint grafting provided an additional opportunity for fusion, and in some extreme situations, it was the sole grafting bed available.

To our knowledge, this is the first study to delineate the necessity of structural bone grafting in atlantoaxial facet joints. The present study suggested that for those without BI, inserting a strut graft into the facet joint space gained no additional benefit for successful maintenance of the reduction or the clivus-axial angle correction. For those without BI, the height of the facet joint space was less than 3–5 mm following cartilage removal. Forcefully inserting a spacer into such a limited joint space seems unnecessary, although our study showed that it did not lead to iatrogenic harm. Structural grafting significantly increased the postoperative height of the atlantoaxial lateral mass joint space and maintained it better than nonstructural grafting. However, the clinical contribution of the height increase was in doubt for those with normal lateral mass column height.

For those with BI or facet column shortening or collapse due to any etiology, a large, “natural” intraarticular space could be acquired by skull traction, the release of atlantoaxial joints and manual reduction. In the present study, heights ranging from 5 to 11 mm were acquired. Inserting a spacer into such ample space is not technically challenging. Moreover, structural grafting provided biomechanical advantages in such situations, which was akin to the basic principle of anterior cervical discectomy and fusion. Vertical, sagittal, and rotatory stress could be restrained by rigid fixation and the use of strut grafts within the facet joint space. The present study showed that maintaining postoperative reductions in BI patients was more successful with structural grafting than nonstructural grafting (*P*<0.05). Combined with the intraoperative feasibility the technique provides to surgeons, the results suggested that structural grafting was strongly advocated in cases of facet spaces greater than 5 mm following vertical reduction. Moreover, structural grafting significantly increased the postoperative height of the joint space and maintained it much better than nonstructural grafting (*p* < 0.05), which contributes much to BI correction. We uniformly adopted autogenous tricortical iliac crest bone as the spacer in the present study, as it is regarded as the gold standard among bone grafts, and the intraarticular joint space was not regularly shaped enough for a cage or mesh.

Our study had certain limitations. The case series involved a small cohort. Moreover, the asymmetry of patient allocation in the nonstructural and structural grafting groups with a ratio of 2.7:1 limited comparison between groups. The biomechanical advantages of structural grafting were inferred from theoretical presumptions based on experience in similar situations in other spine regions instead of on rationale derived from a biomechanical study. Most importantly, one shortcoming of the present study is that we could only suggest a range instead of an accurate height value for the atlantoaxial facet joint space as a criterion for grafting measure selection, and a prospective clinical trial with a larger sample and biomechanical test is required to obtain such a value.

## Conclusion

Intraarticular structural bone grafting in atlantoaxial facet joints has the advantage of maintaining anterior column height in the case of lateral mass collapse or when BI correction is needed; otherwise, nonstructural bone grafting should be considered.

## Supplementary Information

**Additional file 1: Figure S1.**

**Additional file 2: Figure S2.**

**Additional file 3: Figure S3.**

## Data Availability

The data and materials might be obtained from the corresponding author upon request.

## Abbreviations

BI: Basilar invagination
NBI: No basilar invagination
S: Structural grafting
NS: Non-structural grafting
Preop: Preoperation
Postop: Postoperation
LF: Last follow-up
