# Supplementary figures and images for: Intraarticular bone grafting in atlantoaxial facet joints via a posterior approach: nonstructural or structural—a minimum 24-month follow-up
Source: J Orthop Surg Res. 2021 Aug 23;16:524. doi: 10.1186/s13018-021-02630-z (PMC8381565; doi:10.1186/s13018-021-02630-z)

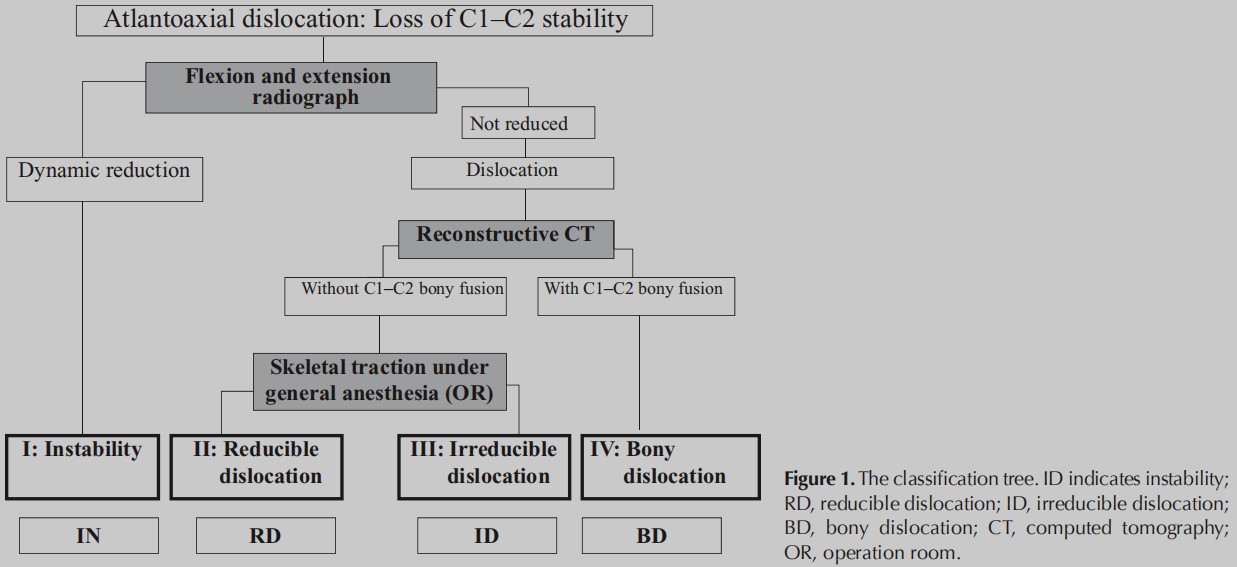

Supplement: Supplementary file 1 — Additional file 1: Figure S1. [file 13018_2021_2630_MOESM1_ESM.jpg]

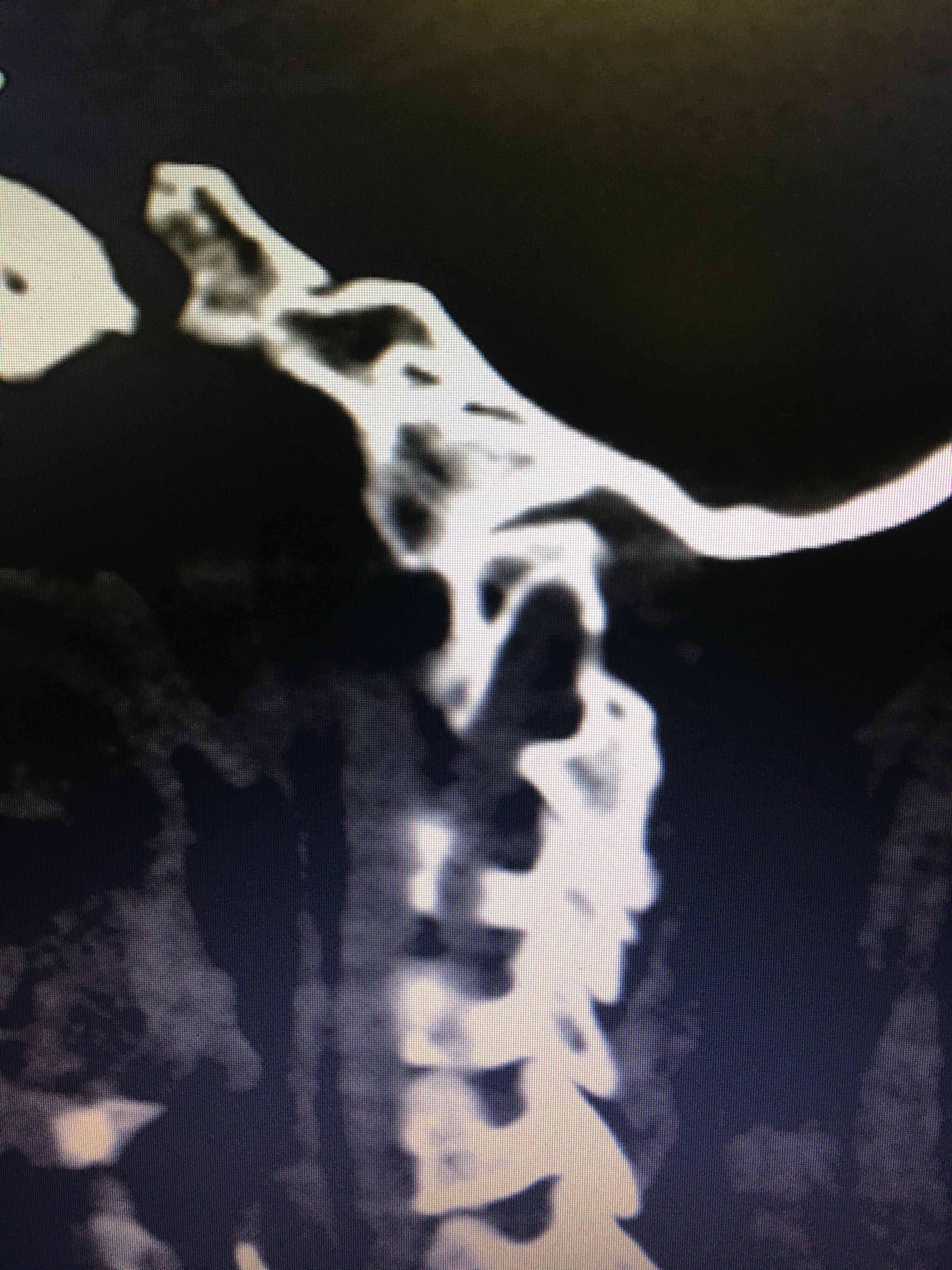

Supplement: Supplementary file 2 — Additional file 2: Figure S2. [file 13018_2021_2630_MOESM2_ESM.jpg]

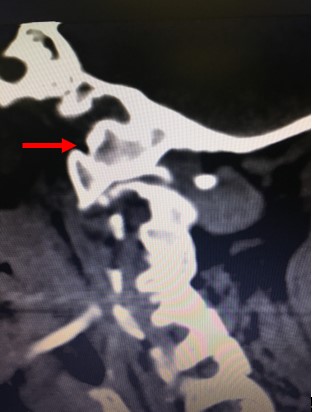

Supplement: Supplementary file 3 — Additional file 3: Figure S3. [file 13018_2021_2630_MOESM3_ESM.jpg]
